# The influence of age on the match-to-match variability of physical performance in women’s elite football

**DOI:** 10.3389/fphys.2023.1193501

**Published:** 2023-05-15

**Authors:** Ivan Baptista, Andreas K. Winther, Sigurd Pedersen, Dag Johansen, Svein Arne Pettersen

**Affiliations:** ^1^ Department of Computer Science, Faculty of Science and Technology, UiT The Arctic University of Norway, Tromsø, Norway; ^2^ Faculty of Sport, Center of Research, Education, Innovation in Sport (CIFI2D), University of Porto, Porto, Portugal; ^3^ School of Sport Sciences, Faculty of Health Sciences, UiT The Arctic University of Norway, Tromsø, Norway

**Keywords:** high-intensity actions, locomotor demands, playing position, peak periods, player experience

## Abstract

**Introduction:** The fluctuation of external match load throughout a season is influenced by several contextual factors. While some, have been deeply analysed in men’s football literature, information is lacking on how other contextual elements, such as player’s age or experience, may affect the match-to-match variability of locomotor activities. In fact, aging has been described as a multifactorial process with the potential to affect human performance. The aim of this study is to assess if the variability of match locomotor performances fluctuates according to the players’ age.

**Methods:** 59 female players from four top-level clubs were divided into three age groups and monitored during two seasons using GPS APEX (STATSports, Northern Ireland), with a sampling frequency of 10Hz, in 150 official matches to determine the coefficient of variation (CV) of full-match and 1-min peak locomotor demands of total distance (TD), high-speed running distance, sprint distance (SpD), accelerations, and decelerations. To test whether there was a group effect of age on match-to-match variability we used a one-way ANOVA with CV% as the independent variable.

**Results:** CV values of full match variables ranged from 3.8% to 27.8%, with total distance (3.8%) in the peak age group and SpD (27.8%) in the pre-peak age group. Similarly, CV values of 1-min peaks ranged from 4.1% (post-peak group) in TD to 22.3% (peak group) in SpD.

**Discussion:** The main finding was that there were no significant differences between the different age groups in the metrics analysed although trends indicate less variability in the post-peak age group.

## 1 Introduction

The daily monitoring of football players has become common practice in professional and semi-professional environments (Al Haddad et al., 2018). Researchers have used quantitative data captured to provide practitioners with novel insights into training (Akenhead et al., 2016) and match demands (Baptista et al., 2019). During the past decade, evidence-based insight concerning the players’ locomotor activity during competition has substantially increased, not only due to the rule change regarding the use of wearable technologies during official matches (FIFA, 2015), but also as a consequence of “new” approaches and perspectives (Kirkendall, 2020; Teixeira et al., 2021).

One hot topic is the degrees of variability associated with the players’ physical performances across consecutive matches. Recent studies have revealed the importance of analysing the match-to-match variability of locomotor activities (expressed by the coefficient of variation (CV) of a particular measurement) by presenting its practical utility (Oliva-Lozano et al., 2021; Winther et al., 2022). The fluctuation of external match load throughout a season should be considered part of the nature of team sports (Baptista et al., 2022), and is influenced by many contextual factors. While some of these factors, such as playing position (Bush et al., 2015), seasonal period (Mohr et al., 2003), ball possession (da Mota et al., 2016), opposition (Rampinini et al., 2007), match location (Castellano et al., 2011), and playing style (Oliva-Lozano et al., 2021) have been deeply analysed in the literature in men’s football, information is lacking on how other contextual elements (e.g., player’s experience) may affect the match-to-match variability of locomotor activities especially in women´s football.

Players’ experience may be noted in several match-related behaviors, such as tactical decisions (Buchheit et al., 2010) or even pacing strategies (Lorenzo-Martínez et al., 2020). If team sports players pace their efforts, then it should be expected that more experienced players may have a better knowledge on how their body responds to the match demands and present more stable performances both within and between matches. However, previous findings in football populations showed divergent conclusions (Castagna et al., 2005; Lorenzo-Martínez et al., 2020), suggesting other factors associated with age (e.g., fitness level, the recovery process, *etc.*) may also affect match-to-match variability.

Despite not being the same as experience *per se*, aging has been described as a multifactorial process with the potential to affect human performance (Bruce, 1984; Castagna et al., 2005). This idea is corroborated in recent studies, where players over 30 years of age presented significantly lower total distance, high-speed running, and sprint distance covered than younger male players (Rey et al., 2019; Sal de Rellán-Guerra et al., 2019). Such a finding may be influenced by physical capacity. For example, there is a correlation between the Yo-YoIR1 test and high-intensity running (>18 km h^−1^) (Krustrup et al., 2005), and further, a u-shaped relationship exists between age and the Yo-YoIR1 test in female players (Datson et al., 2022). Furthermore, according to Hauer et al. (2021), older male players might be considered more experienced and tend to play a more tactically demanding game, thus preserving part of their physical capacities. The age range (∼18–38 years of age) of players in professional football teams is considerable (Dendir, 2016), which means differences in fitness level, tactical knowledge, and self-regulated performance are expected to appear within a team. To the best of our knowledge, the only study investigating the age- or experience-related variability of locomotor activities during match-play in professional football players is done by Lorenzo-Martínez et al. (2020). The authors studied Spanish male football teams and observed that younger players (<25.2 years of age) presented lower CVs for high-speed activities compared to older players (>33.1 years of age).

However, as previously reported for weightlifters (McGuigan and Kane, 2004) and cyclists (Paton and Hopkins, 2006), it seems that female athletes present higher CVs than men. Despite playing the same game, reference values for men’s football should not be used for decision-making in women’s football, thus requiring further investigation within this field with female players. Moreover, as several sources of bias are expected when a single team is analysed or when a small sample size is used, a multiple-team analysis is warranted (Gregson et al., 2010). Therefore, due to the lack of evidence in team sports in general and in women’s football in particular, the aim of this study is to assess if the variability of match locomotor performances (full match and peak demands) expresses different magnitudes according to the players’ age. We hypothesize that younger players present a minor/less pronounced variability than older players.

## 2 Methods

### 2.1 Participants and match samples

With ethical institutional approval from the Norwegian Centre for Research Data (reference number: 296155) and written informed consent from the participants, physical performances of 59 players from 150 official matches during two entire seasons (2020 and 2021) in four top level clubs were collected using GPS APEX (Statsports, Northern Ireland), with a sampling frequency of 10 Hz. The validity and accuracy levels (bias <5%) of this tracking system have been previously reported (Beato et al., 2018). Each player used the same GPS unit throughout the season to minimize inter-device error (Beato et al., 2018). Goalkeepers were excluded from analysis, and the playing positions (central defenders, full-backs, central midfielders, wide midfielders, and forwards) were classified according to previous research (Baptista et al., 2022). Match observations under 90 min and observations where players did not play in their main (modal) position were also excluded from the analysis. In addition, only players with three or more match observations were included in the sample. Next, we calculated the coefficient of variation (CV) and mean age for each player before dividing the players into three groups: pre-peak age (<23 years old), peak age (23–27 years old), and post-peak age (>27 years old) (Table 1). Given that peak age studies in women’s football are almost non-existent we based the peak age dichotomization on 1) the study by Barreira et al. (2021) reporting a peak age of approximately 25 years old, and 2) studies on male elite players showing peak age band between 25–27 years old (Dendir, 2016). We also felt extending this age band to include ages 23–25 years was appropriate due to the league being comparatively young in comparison to the men’s league. The final sample characteristics are shown in Table 1. The STROBE cohort reporting guidelines were used according to the recommendations for epidemiological observational studies (von Elm et al., 2014).

**TABLE 1:** Age group distribution according to peak age (mean and standard deviation of age).

Age group	Position	Number of players	Mean age	Std age
Pre-peak	Central Defender	4	19.2	1.1
Pre-peak	Central Midfielder	13	19.6	1.3
Pre-peak	Wide Defender	4	19.9	1.7
Peak	Central Defender	5	24.5	1.1
Peak	Central Midfielder	9	25.1	1.1
Peak	Wide Defender	5	25.9	1.2
Peak	Wide Midfielder	2	23.7	0
Post-peak	Central Defender	5	27.8	0.7
Post-peak	Central Midfielder	3	29.4	1.1
Post-peak	Forward	6	29.7	2.2
Post-peak	Wide Defender	3	29.6	1.6

### 2.2 Physical performance variables

The physical parameters analysed included: total distance (TD), high-speed running distance (HSRD (>4.44 m s^-1^), sprint distance (SpD) (>5.55 m s^-1^), and the number of accelerations and decelerations (Acc/Dec). The speed thresholds were chosen according to previous research (Strauss et al., 2019; Baptista et al., 2022). Accelerations and decelerations were defined as a positive or negative change in speed of more than ±2.26 m s^-2^, with a minimal effort duration of 0.3 s, finishing when the rate of acceleration/deceleration reached 0 m s^-2^. All variables were used to analyse full match (absolute values) and peak locomotor demands (1-min rolling analysis period). The epoch length of 1 min to analyse the peak locomotor demands was chosen to find the highest relative intensities in accordance with previous research (Doncaster et al., 2020).

### 2.3 Statistical methods

Doppler-derived speed data was exported from manufacturer software (StatSport Sonra 2.1.4), and we used Python 3.9.12 for processing (linearly interpolating any missing raw data) and to derive metrics. Raw acceleration was then calculated over 0.6 s. After deriving all the metrics, the data was transferred to R (R.4.1.3, R Core Team, 2022) for statistical analysis. To test whether there was a group effect of age on match-to-match variability we used a one-way ANOVA with CV% as the independent variable. If a significant effect was found (*p* ≤ 0.05), then pair-wise comparisons were done using Tukey’s Honestly Significant Difference.

## 3 Results

Descriptive statistics of full-match and peak period demands of each playing position from the different age groups are presented in Table 2. Across the three playing positions (CD, WD and CM) represented in all age groups, the higher full match values observed in TD, HSRD, SpD, and Acc were from the players in the post-peak age group, with the lowest being observed in the pre-peak age group. When analysing the 1-min peak periods, the CD with the highest TD, HSRD, and SpD were from the peak age group, while for CM the highest values of these metrics were found in the post-peak age group.

**TABLE 2:** Means, standard deviation and coefficient of variation per playing position in the different age groups.

Metric		Pre-peak age group			Peak age group				Post-peak age group			
		CD	WD	CM	CD	WD	CM	WM	CD	WD	CM	FW
TD (m)	Full match	9,028 ± 444	10070 ± 331	10277 ± 656	9,336 ± 216	10298 ± 407	10507 ± 539	10470 ± 729	9,670 ± 479	10390 ± 371	10846 ± 613	9,632 ± 339
	1-min peak	326 ± 17	360 ± 16	395 ± 102	393 ± 144	374 ± 11	356 ± 20	363 ± 27	338 ± 16	376 ± 2	588 ± 320	389 ± 128
	CV (%)	5.6	4.9	4.2	3.6	3.7	4.0	6.7	3.4	4.5	3.5	5.8
HSRD (m)	Full match	969 ± 149	1,472 ± 219	1,555 ± 351	1,027 ± 157	1722 ± 209	1,609 ± 349	1709 ± 699	1,217 ± 229	1905 ± 29	1922 ± 632	1,417 ± 230
	1-min peak	123 ± 13	160 ± 19	165 ± 50	146 ± 56	175 ± 16	149 ± 24	158 ± 32	132 ± 16	182 ± 12	248 ± 125	151 ± 44
	CV (%)	21.0	12.9	13.9	16.5	11.1	14.7	18.7	16.0	12.9	15.0	12.9
SpD (m)	Full match	227 ± 40	353 ± 118	378 ± 154	222 ± 70	472 ± 78	381 ± 172	382 ± 269	281 ± 90	528 ± 142	385 ± 197	352 ± 95
	1-min peak	63.5 ± 7.6	78.3 ± 18.6	85.2 ± 32.7	68.6 ± 24.2	94.0 ± 8.6	71.6 ± 18.3	73.4 ± 24.3	68.0 ± 11.3	102.0 ± 22.2	112.5 ± 60.3	78.4 ± 24.9
	CV (%)	38.2	23.5	29.3	31.5	20.7	27.9	31.0	32.3	16.3	28.2	22.0
Acc	Full match	385 ± 45	423 ± 75	482 ± 113	430 ± 54	487 ± 56	488 ± 111	475 ± 141	464 ± 160	572 ± 83	525 ± 91	485 ± 77
	1-min peak	47.8 ± 2.6	50.9 ± 3.4	60.7 ± 22.9	57.6 ± 22.5	58.9 ± 2.5	52.2 ± 8.1	51.5 ± 8.2	51.7 ± 11.4	60.6 ± 7.1	78.2 ± 36.0	59.7 ± 19.6
	CV (%)	12.7	15.3	12.8	12.8	11.1	14.6	18.6	13.7	12.6	12.1	13.9
Dec	Full match	309 ± 43	360 ± 99	438 ± 98	295 ± 43	432 ± 33	391 ± 99	421 ± 167	353 ± 93	470 ± 57	404 ± 48	370 ± 62
	1-min peak	36.5 ± 3.6	38.7 ± 6.1	46.4 ± 15.3	41.9 ± 17.5	44.2 ± 3.4	39.4 ± 6.2	42.7 ± 8.2	36.4 ± 7.0	43.7 ± 6.0	62.1 ± 32.6	43.8 ± 13.2
	CV (%)	14.0	13.5	15.4	16.0	8.2	12.7	17.5	12.0	13.2	15.7	11.8

CV, coefficient of variation.

The variability of full match and 1-min peak locomotor demands per age group is presented in Table 3. All estimates of match-to-match variability from the three age groups analysed (pre-peak, peak, and post-peak) are expressed in percentage (CV) units. CV values of full match variables ranged from 3.8% to 27.8%, with the lowest CVs appearing in the TD of the peak age group (3.8%) and the highest in the SpD of the pre-peak age group (27.8%). Metrics of 1-min peaks presented similar values with the CV ranging from 4.1% (post-peak group) in TD to 22.3% (peak group) in SpD. For every age group analysed, the metrics with the lowest and the highest degrees of variability were TD and SpD, respectively. Post-peak age group presented the lowest full match CVs for HSRD (13.3%), SpD (22.1%), and Acc (12.6), while the pre-peak age group presented the highest full match CVs for TD (4.2%), SpD (27.8%), and Dec (13.8%). Conversely, the highest 1-min peak CV for TD (5.0%), HSRD (14.0%), SpD (22.3%), and Dec (13.6%) were observed in the peak age group. The only metric where the highest CV was observed in the post-peak age group was the 1-min peak Acc (14.4%), with all the others presenting their highest values both in the pre-peak or in the peak age groups. However, no significant differences were found in any metric analysed between the different age groups.

**TABLE 3:** Match-to-match variability of full match and 1-min peak demands expressed as coefficients of variation (%).

Metric		Match-to-match variability - CV (90% CI)^a^			Post-hoc comparisons (*p* < 0.05)
		Pre-peak age group	Peak age group	Post-peak age group	
TD	Full match	4.2 (3.5–5.0)	3.8 (3.1–4.5)	4.1 (3.3–4.9)	No sig. Differences
	1-min peak	4.7 (3.7–5.6)	5.0 (4.0–6.0)	4.1 (3.3–4.9)	No sig. Differences
HSRD	Full match	14.0 (11.8–16.2)	14.1 (11.8–16.3)	13.3 (10.9–15–7)	No sig. Differences
	1-min peak	13.7 (11-9–15.5)	14.0 (12.2–15.8)	12.4 (10.7–14.0)	No sig. Differences
SpD	Full match	27.8 (22.6–33.0)	26.1 (21.2–31.0)	22.1 (17.5–26.7)	No sig. Differences
	1-min peak	21.2 (17.6–24.9)	22.3 (18.7–25.9)	21.9 (18.3–25.6)	No sig. Differences
Acc	Full match	12.8 (10.9–14.6)	13.1 (11.2–14.9)	12.6 (10.6–14.6)	No sig. Differences
	1-min peak	12.5 (10.3–14.7)	14.3 (11.9–16.7)	14.4 (11.9–16.9)	No sig. Differences
Dec	Full match	13.8 (11.8–15.8)	12.3 (10.5–14.0)	12.4 (10.5–14.4)	No sig. Differences
	1-min peak	12.9 (11.0–14.8)	13.6 (11.7–15.5)	12.7 (10.8–14.5)	No sig. Differences

CV, coefficient of variation; CI, confidence intervals.

^a^ Values presented as percentage of the mean.

## 4 Discussion

This study is the first attempting to analyse potential influence of age on the match-to-match variability of physical performances in elite women´s football. Although presenting trends indicating less variability in the post-peak age group, the main finding was that there were no significant differences between the different age groups in the metrics analysed. Causality of performance fluctuations related to age may have some intricate aspects that are worth reflecting over. One aspect is how player experience impacts his/her own energy expenditure over a game, e.g., pacing strategies based on experience spreading the expected load over 90 min (Paul et al., 2015). Another aspect may be related with the coaches’ strategies, where style of play and selection of players for more demanding positions might take the age factor into account. However, this hypothesis was not confirmed in the present study, with the results revealing no significant differences between the age groups in any of the analysed parameters. Our results suggest that, either age has a small impact in the variability of match physical performance, or that other contextual factors may have a higher influence, masking eventual differences. Opponent’s level, for instance, is one of these contextual factors that might have influenced the outcomes of the study (Rampinini et al., 2007), since we did not control if there were discrepancies in the level of the opponents across age groups. Furthermore, the non-significant differences observed between age groups can also be a consequence of the dynamic and stochastic nature of football matches, which makes it hard to predict or to establish a rationale behind the players’ physical performances (Baptista et al., 2022).

Surprisingly, when analysing the differences in match physical performance between the different age groups, we observed that the highest values in the various parameters were performed by the post-peak age group. This evidence contrasts with previous research on male players where more experienced players performed significantly lower TD, HSRD, and SpD than less experienced players (Rey et al., 2019; Sal de Rellán-Guerra et al., 2019). While in specific playing positions (i.e., CM) these results may be biased by the considerably lower number of subjects allocated in the post-peak age group (3) than in the pre-peak (13) and peak (9) age groups, in the other playing positions (CD and WD) such differences in the sample size were not presented. Another possible explanation, for the above-mentioned differences between studies, is that there might be a sex difference in the variability of match physical performance between the sexes. On the other hand, it seems to exist a higher heterogeneity in the 1-min peak variables, where no trend was observed across age group performances. Altogether, these results might reveal that, in women’s football, age is not a limiting factor for match performance and that older players can equally cope with the peak match demands as the younger players do.

### 4.1 Limitations and further research

In order to increase data heterogeneity and diminish the risk of bias, we followed the suggestion of previous research (Al Haddad et al., 2018; Oliva-Lozano et al., 2021) for the necessity to conduct multi-club studies, by including four different top-level teams. However, the peak age calculation and the age group distribution resulted in different number of players per playing position and an unequal number of playing positions per age group. This may be seen as a limitation of the study since previous studies have reported different CVs across positions (Gregson et al., 2010; Bush et al., 2015; Carling et al., 2016; Al Haddad et al., 2018). For instance, the pre-peak age group was represented only by players from three playing positions (CD, WD and CM), while WM and FW were only included in the peak and post-peak age group, respectively.

Furthermore, in the present study only univariate peak locomotor demands were considered in the analysis of the peak periods’ variability, which may have influenced the results obtained. Further research within this field should attempt to include multivariate peak periods in their analyses. Moreover, a direct comparison with male football should be interpreted with caution since peak age cut-offs of female and male football players are expected to be different. Finally, one of the challenges in research with female top-level football clubs is the size of the samples available. Therefore, a statistical type-2 error should not completely be ruled out.

## 5 Conclusion

Differences in the match-to-match variability of physical performances between age groups in women´s elite football are probably small. Although no significant differences were revealed in the present study, the influence of age in the players’ ability to maintain stable physical performance across matches seems to be logical and reasonable and should be deeper studied in further research, where the study limitations here presented may be considered in study designs. Despite an eventual influence on the players’ capability to pace their energy expenditure during and across matches, age seems to not be a limiting factor for peak match performance.

## Data Availability

The raw data supporting the conclusions of this article will be made available by the authors, without undue reservation.
